# Evaluation of Placental mir-155-5p and Long Non-coding RNA sONE Expression in Patients with Severe Pre-eclampsia

**Published:** 2017-01-17

**Authors:** Faezeh Azizi, Soraya Saleh Gargari, Sedigheh Asadi Shahmirzadi, Fatemeh Dodange, Vahid Amiri, Reza Mirfakhraie, Mir Davood Omrani

**Affiliations:** 1 *Department of Medical Genetics, Faculty of Medicine, ShahidBeheshtiUniversity of Medical Sciences,Tehran, Iran.*; 2 *Feto-Maternal Unit, ShohadayeTajrish Hospital, Shahid* *Beheshti, University of Medical Sciences, Tehran, Iran.*; 3 *Feto-Maternal Unit, Mahdiyeh Hospital, Shahid* *Beheshti, University of Medical Sciences, Tehran, Iran.*; 4 *School of Allied Medical Sciences,* * Shahid* *Beheshti, University of Medical Sciences, Tehran, Iran.*

**Keywords:** Preeclampsia, miR-155-5p, long non- coding RNA sONE, real time PCR

## Abstract

It has been well documented that preeclampsia (PE) has a common etiological background, but little is known about its linkage at the molecular level.Non- coding RNAs are critical posttranscriptional regulators ofgene expression. This study was performed to determine whether PE is associated with alterations in placental non-coding RNAs expression. MicroRNA (miR)-155-5p and long non-coding RNA (*lnc*)*sONE* expression, in placentas collected sequentially from 59 patients with PE and 40 normotensive pregnancies were measured using real-time PCR.The relationship between miR-155-5p and *lncsONE* expressions was analyzed statistically. miR-155-5p expression was increased (fold change =1.6, P=0.04), while lncsONE expression was not significantly changed (fold change =1.1, P=0.68), in placentas from patients compared with control group.miR-155-5p was upregulated in placentas from patients with PE and may have influenced *eNOS* expression. These findings indicate that miRNA-155-5p may be involved in PE pathogenesis and could be a potential biomarker for this disease.

Despite remarkable progress in the prenatalogy science, pre-eclampsia (PE), a syndrome affecting 5-7% of pregnancy disorders, still remains as the most prevalent cause of maternal and fetal morbidity and mortality (1). According to the WHO definition, PE is a pregnancy-related syndrome characterized by hypertension (greater than or equal to 140/ 90) and proteinuria (greater than or equal to 300 mg in a 24 h urine specimen) following 20 weeks of gestation (2). Based on the elusive pathophysiology, PE has traditionally been called the disease of theories (3, 4). Although, the association between the pathology of PE and different factors, such as genetic pathways polymorphisms and expression like Renin–angiotensin, angiogenesis, coagulation factors (methyl tetrahydrofolate gene and V-leiden), free radical metabolism networks, and genes related to placentation, has been evaluated in numerous studies, unfortunately, none of these investigations were successful to introduce a main factor for the disease (2, 5). Since the only way to treat PE is termination of pregnancy, hence the presence and the function of the placenta in development of PE is considerable and therefore it can be a main clue of changes in PE (5). In order to prevent and treatPE, numerous experiments have been performed in both animal and human models using NO donors such as organic nitrates and NO precursors like L-Arginine (5, 6). Production and function of NO is important in normal and PE pregnanciesfor the following reasons:i) Dysfunction of placenta endothelium, as a main initiating mechanism,may occur as a result of oxidative stress, which is characterized by decreased NO production and hypoxia. ii) It has been indicated that the production level of superoxide anion and other alternative metabolites of NO production pathway, including ornithine and asymmetric dimethylarginine (ADMA) is increased in the serum of patients, which could be considered as an early stage biomarker for the disease. iii) Several novel therapeutic approaches are affecting NO production pathway (4,6,7). In endothelial cells, NO is encoded by *eNOS3*, which is negatively regulated through a multitude of molecules, including non- coding RNAs (mir-155) and lncRNA*sONE*. miRNA, single strand and ~22- nucleotide- long non- protein coding RNAs, are able to regulate gene expression by destabilizing the mRNA or down-regulating their target genes (8). Mir- 155 is the product of the polygenic conserved region of B- cell integration cluster gene, which is composed of 3 exons and located in chromosome 21q21 (9). Evidences indicated that mir-155 was up-regulated in the collection of placenta from a large number of pregnant women suffering from PE. Indeed, *eNOS *is considered to be one of the main targets of mir-155. It is well- established that over-expression of miR-155can lead to decrease of*eNOS* expression (10, 11). Moreover, several studies indicated that post translational alteration of sONE, is one of the mechanisms involved in *eNOS *regulation. *sONE*is an anti-sense RNA that is oriented in a tail-to-tail configuration next to *NOS3*. The region of*sONE* and *eNOS* have 662 nucleotides overlap with each other and *sONE* gene covers the 3' UTR region of *eNOS* mRNA (12). Since the production of NO by*eNOS3* is pivotal in different stages of the development of the placenta, such as invasion of trophoblast cells and angiogenesis, it is reasonable to hypothesize that up-regulation of *sONE* could be one of the main reasons for down-regulation of *eNOS3 *(13). Given these, we aimed to evaluate the expression level of mir-155-5p and *lncone* in the collections of placental tissues of PE women and compare with placental tissues of normalpregnant women.

## Materials and methods

**Study population**

Placental tissue samples were sequentially collectedfrom 59 Iraniannulliparous pre-eclamptic womenwithout family history of disease, and 40 gestational age-matched nulliparous normotensive pregnancies (controls) at the Department ofObstetrics and Gynaecology, Mahdiyeh hospital, of ShahidBeheshti University of Medical Sciences, Tehran, Iran during March 2014 and July 2015 after delivery.The average of pre-eclamptic women and control group who delivered by caesarian sections were 70% and 12.5%, respectively. PE was defined according to the American College of Obstetricians andGynecologists guidelines as gestational hypertension (systolic pressure **>**140mmHg and /or diastolic bloodpressure> 90mmHg on at least two occasionsafter gestational week 20), with proteinuria (>0.3 g/24 h).Severe PE was defined as blood pressure above 160 mmHg systolic and/or 110 mmHg diastolic or proteinuria >5 g/24 h or oliguria (<500 ml urine/24 h). All pregnancies were otherwise uncomplicated singleton pregnancies except for the development of PE. Patients with chronic hypertension, renaldisease, collagen vascular disease,a positive family history for PE and otherpregnancy complications (such as fetalanomalies or chromosomal abnormalities) were excluded from this study. The EthicsCommittee of ShahidBeheshti University of Medical Sciences approved the consentforms and procedures necessary to utilizethe tissues, and written informed consentwas obtained from all participants prior tosurgery.

**Placental biopsy collection**

Full-thickness blocks of 3–5 cm were taken from the middle region of the placenta. A central area of chorionic tissue was dissected, and the maternal deciduas and amnionic membranes were removed. After vigorous washing of the maternal blood with saline solution, tissues were immediately frozen in liquid nitrogen and stored until use. Collected tissue samples were washed with 1 × PBS to remove contamination of maternal blood, placed immediately into RNAlater solution (AmbionInc, Life Technologies) and kept at −80 °C until RNA isolation. All samples were collected by the same medical personnel and using identical protocol.

**RNA extraction**

RNA was extracted using the miRNeasy Mini Kit (Qiagen Inc, Valencia,CA) according to manufacturer’s instructions. RNA quality was assessed by agarose gel electrophoresis. Only samples with two clearly detectable ribosomal RNA bands (28S and 18S) and a higher than 2:1 ratio-were evaluated by nanodrop for purityof intact RNA, and were used for reverse transcription.

**cDNA synthesis and quantitative real- time reverse transcription (RT) PCR for miR-155 expression analysis**

**Fig. 1:**
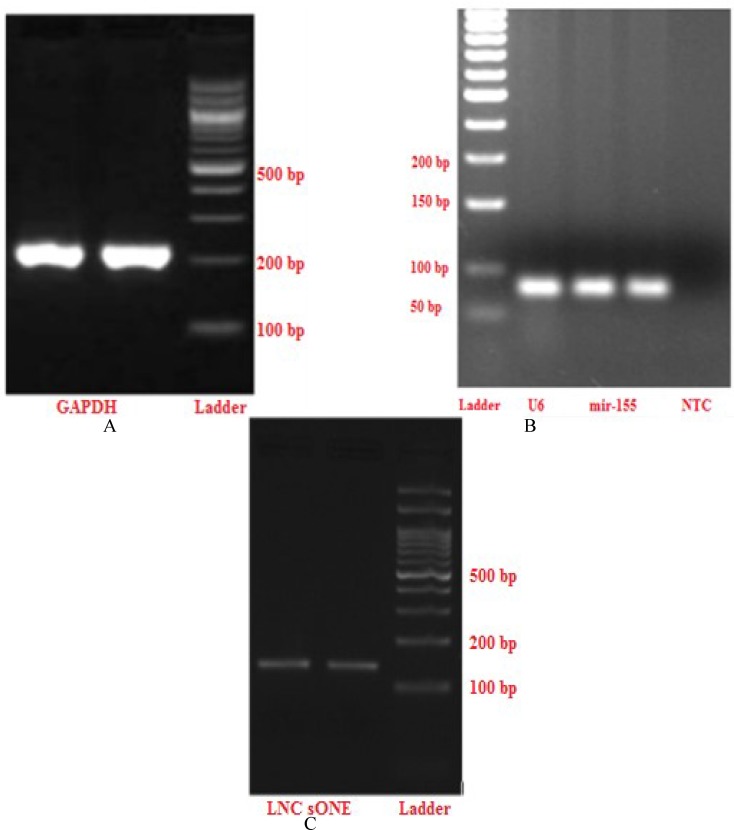
Electrophoresis of real time PCR products. A: electrophoresis of real time PCR products for the *U6* and miR-155 genes at 75 and 78 bp, respectively. Lane 1 is the molecular weight marker (50 bp DNA ladder), lane 2 is the reference gene of U6, lanes 3 and 4 are miR-155 and lane 5 corresponds to non template control (NTC). B: electrophoresis of real time PCR products for the *GAPDH* gene at 206 bp. Lane 1 and 2 show bands of target gene, lane 3 is the molecular weight marker (100 bp DNA ladder). C: electrophoresis of real time PCR products for *lncsONE* gene at 146 bp. Lane 1 and 2 show bands of target gene, lane 3 is the molecular weight marker (100 bp DNA ladder

Expression of miR-155was assessed byquant-itative real-time RT–PCR(SYBR green method), and thesmall nuclear RNA U6was used as aninternal control. The miR-155 and RNAU6-specific cDNA sequences were synthesizedfrom total RNA using gene-specificprimers (Table 1). cDNA synthesis was performed by reverse specific primers by a method referred to as adapter method. At first, reverse transcription reactions contained7.5 µltotal RNA,0.5 µl Poly adenine polymerase (PAP)(NEB# M0276L,USA), 1µl PAP Buffer (10x)and 1µl dNTPs (10 mM). The 10□l reaction mixture was initiallyincubated at 37°C for 20-30 min, then 65°C for 20 min. After this step, Thermo Scientific kit (K1622, USA) was used by adding 2 µlRT Adaptor to 5 µl template RNA synthesizedduring the first step, 5 µl nuclease free water,1µlRiboLock RNase Inhibitor (20 U/□l), 2 µl dNTPs (10mM) and1 µlRevertAid M- MuLV RT (200 U/ΜL).Then the mixture was incubated at 25°C for 5 minat 42°C for 1h and finally70°C for 5 min. After performing gradient PCR to find the appropriate temperature (Figure 1. A), standard curve was plotted for each primer till final analysis was done according to a Livak^,^s formula (2^^-Δct^).Real-time quantitative PCR was performe-dusing a Corrbetreal-time PCR detection system (Rotor gene, Corbett 6000(2.1.0), Qiagen, Germany). Each 15μl reaction volume contained7.5 µl of master mix (RR820B, Takara), 5 µl nuclease free water, 1µl offorward primer plus universal reverse and1.5µl cDNA. PCRamplifi-cation of mir- 155 was initiated at 95°C for 5 min, followed by 45 cycles of 95°C for15 s, 61°C for 45s.

**Table 1:** Sequences of used primers

**Annealing temprature**	**Length/bp**	**Sequence**		**Gene name**
61°C	146	5' -CCCCTCATACAAGAAGCTCCC-3'	F	*lnc sONE*
		5' -TGCAGGTTGAGCCTGTGTTG-3'	R	
64°C	206	5' -AGGGCTGCTTTTAACTCTGGT-3'	F	*GAPDH*
		5' -CCCCACTTGATTTTGGAGGGA-3'	R	
61°C	78	5' -GAGGGTTAATGCTAATCGTGATAGG-3'	F	miR-155-5p
		5' -GCACAGAATCAACACGACTCACTAT-3'	R	
61°C	75	5'-CGCAAGGATGACACGCAAATTC-3'	F	*U6*
		5' -GCACAGAATCAACACGACTCACTAT-3'	R	
		5' –GTCGAGCACAGAATCAACACGACTC-3' ACTATAGGTTTTTTTTTTTTTTTVN-3'		RT adaptor

**Table 2:** Distribution of the selected variables between the severe PE cases and control subjects

	**Preeclampsia (n=59)**	**Control (n=40)**	**P-value**
Age (years)	27.42 ± 6.7	23.78±4.15	0.15
BMI at delivery (kg/m^2^)	25.90 ± 3.96	23.33±2.53	<0.001
SBP (mmHg)	150.33±17.66	108.75±7.23	<0.001
DBP (mmHg)	94.85±10.13	71.63±5.11	<0.001
Urinary protein (g/24 h)	1.55±0.77	absent	-
Gestational age at delivery (weeks)	37.13±2.95	38.75±1.10	<0.001
kind of delivery Normal Cesarian	- 30% 70%	- 87.5% 12.5%	- - -

BMI: body mass index; SBP: systolic blood pressure; DBP: diastolic blood pressure. Data are presented as means ± SD with P <0.05

**cDNA synthesis and quantitative real-time reverse transcription (RT) PCR for** ***lncone*** **expression analysis**

Following total RNA extraction, *lncone*RNA was analyzed by converting 5 µl RNA sample to cDNA for each sample, using an RNAreverse transcription kit with random hexamer and oligo dTprimers (Thermo Scientific kit, K1622, USA), according to the manufacturer’s instructions. Briefly, reagents were prepared to obtain a final concentration of 1 µl random hexamerprimer,1 µl oligo dTprimer, 5 µl template RNA, 5 µl nuclease free water, 1µl RiboLock RNase Inhibitor (20 U/μl), 2 µl dNTPs (10 mM) and 1 µl RevertAid M-MuLV RT (200 U/μl) in a finalvolume of 20 µl.The RT reaction was performedby incubating the samples for 5 minat 25°C, 1 h at 42°C and 5 min at 70°C. Real-time quantitative PCR was performed using Corbet Real-Time PCR System (Rotor gene Corbett 6000(2.1.0), Qiagen, Germany) and SYBR_qPCR Mix (Takara). After performing gradient PCR to find the appropriate temperature (Figure 1. band C) for each primer, standard curve was plotted till final analysis was done according to Livak^,^s formula (2^^-ΔΔCt^). Eacc 5μl reaction volume contained 7.5 µl master mix (RR820B, Takara), 5 µl nuclease free water, 1µl forward and reverseprimers (Table 1) and 1.5µl cDNA. The real-time program for *lncone* was initiated at 95°C for 2 min, followed by 40 cycles of 95°C for 20 s, 61°C for 15 s and 72°C for 20s. The same program was performed for GAPDH except for the annealing temperature that was 64°C.

**Statistical analyzes**

The threshold cycle (Ct) was defined as the fractional cycle number at which Fluorescence passed the fixed threshold. Each sample was measured in duplicate, and the relative amount of miR-155 to U6 and *lncone* to *GAPDH* were calculated using the equation 2^^-ΔΔCt^, where ΔCt = (Ct ^target ^- Ct ^reference^).Statistical analyzes were performed using SPSS 21.0 for Windows (SPSS Inc., Chicago, USA) and Graph Pad Prism 5.0 (GraphPad Software Inc., La Jolla, USA). Data are expressed as mean± SD or as mean ± SEM where appropriate. Comparisons of the groups were examined by Mann–Whitney U test for nonparmetric data. A P value <0.05 was considered as statistically significant for all analyzes.

## Results

**Patients**

In this study the average age and body mass index (BMI) in PE woman were: 27.42 ± 6.7 years, and 23.87 ± 4.15, respectively, and 25.90 ± 3.96 years, and 23.33 ± 2.53, respectively for control group. No significant differences were identified between the normal pregnant and the PE patients with respect to maternal age, but the P- value for BMI was significant (P<0.001) (Table 2).

**Placental expression of miR-155-5p and** ***lncone***

**Fig. 2:**
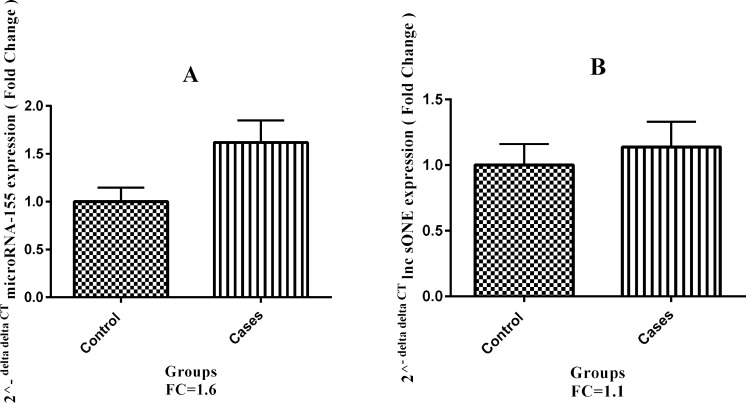
Schematic of the fold changes of mir-155-5p and *lncsONE*. A: mir-155-5p expression change between the PE (n=59) and control (n= 40) groups (mean ± SEM: 4.71±0.24, 5.55±0.34, respectively. By examining 2 ^^ - ΔΔct^ for each patient separately (per case), it was found that, in 74.6% of patients, the expression of mir-155-5p was more than one. B: *lncsONE *expression change between the PE and control groups (mean ± SEM: 4.48±0.20, 4.45±0.23, respectively). By examining 2 ^^ - ΔΔct^ for each patient separately (per case), it was found that, in 47.5% of patients, the expression of *lncsONE* was more than one. FC: fold change

**Fig. 3:**
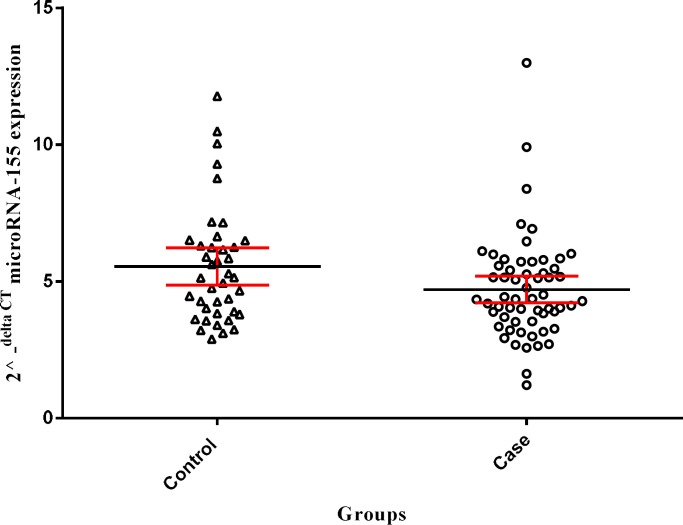
Comparison of the expression patterns of miR-155. miR-155 expression was compared between 59 preeclampsic and 40 normal placentas. A line within the scatter plot marks the median with CI 95%.Whiskers (error bars) above and below the scatter plot indicate the 90th and 10th percentiles (*P ≤ 0.04

Increases in miR-155-5p expression in placental tissue from patients with PE (mean± SEM, 4.71 ± 0.24) was 1.6 fold and was found to be significantly higher than the normotensive control group (mean± SEM, 5.55± 0.34; P=0.04; Figure 2. A and Figure 3).

The expression of*lncone* expression in placental tissue from patients with PE (mean± SEM, 4.48±0.20) was 1.1 fold and was not significantly different from the normotensive control group (mean ± SEM, 4.45 ±0.23;P=0.68; Figure 2.B).

## Discussion

Despite multitude studies, the etiology of pE remains unknown (1). NO regulates a broad spectrum of physiological and cellular processes in several tissues. Based on its multi-functional properties, different therapeutic approaches using NO donors are now under investigation. NO deficiency during pregnancy is associated with high blood pressure and PE, and to date, it seems that NO donors and blockers of NOS inhibitors may bring remarkable advantages for pregnant women. It is hypothesized that in endothelial and placental tissues, aberrant expression of genes which are affecting NO metabolism may be one of the main characteristics of the disease.In this survey, we found that the expression level of mir-155-p had been increased in patients compared to the control group. A previous studyreported that there was a correlation between mir-155, PE that is an excessive inflammation syndrome between mother and fetus response to pregnancy (9). So far, multitude articles have been published that emphasized on the relation between over 100 miRNAs and PE. According to the study conducted by Lycoudi et al. in 2015, it has been demonstrated that aberrant up-regulation of the expression level of miR-210, miR-155 and miR-29b coupled with down-regulation of miR-204, miR-195 and miR-1 were associated with PE (11). Consistently to our results, Li et, al. in 2014, indicated that higher expression level of miR-155 and lower level of *eNOS *expression were detectable in 19 pre-eclampsic placentas compared with 22 normal placentas. Therefore, they suggested thatpresum-ablymiR-155 inhibits the expression of *eNOS* in trophoblast cells. Moreover, they showed that miR-155 regulates the invasion of trophoblast cells through several mechanisms (10). In 2007, a miRNA expression profile in 9 PE placenta was provided by Pineles et al. whoshowed that, 7 different miRNAs had higher expression levels. Among these,miR-155 has been defined as an inflammation-related miRNA, based on the fact that miR-155 can be significantly up-regulated by TNF and lipopolysaccharide (LPS) (15). In another study, Zhang et al. showed that the up-regulation of miR-155 suppressed cysteine rich angiogenic inducer 61 (*CYR61*) in PE placenta, which may be an important pathway in the development of PE. Moreover, miR-155, as a common target of a wide range of inflammatory mediators, can be up- regulated by different inflammatory factors, including LPS, TNF and IL-1 through affecting Toll like receptors (TLR) (9). In a review article, Santa et al. introduced miR-155 as an angiomiR, which is over-expressed in placenta and plays an essential role in pathogenesis of PE (16). In the present study, our results showed that up-regulation of miR-155 couldinfluence the production of NOS in endothelial cells by targeting *eNOS*. Our results suggest that probably miR-155 regulates the placentation hemostasis through modulating the expression level of *eNOS*, becauseas mentioned, miR-155 can inhibit *NOS*mRNA by binding to its 3'UTR.

In our primary analysis, we found that the expression level of *lncone* was sligthly increased. However, this increase was not statistically significant. In 2004, Robb et al. evaluated the expression of *sONE* in human umbilical vein endothelial cells (HUVEC). They found that the expression level of *sONE* was increased in vascular smooth muscle cells upon knock-down of* sONE *by siRNA (17). Moreover, Fish et al. demonstrated that although in the normoxic condition, the expression level of *sONE* was reduced in endothelial cells, in different types of cells under the intense hypoxic condition, the expression level of this mRNA increased significantly. In endothelial cells and normal oxygen condition, a complex of proteins stabilized the *eNOS*mRNA through binding to the *eNOS* 3'UTR (18). It is believed that *eNOS* and *sONE* compete with each other for binding to RNA stabilizing proteins or* sONE/eNOS* RNA interaction prevents the formation of *eNOS* 3'UTR protective complex (18-20). These two hypotheses are under investigation.To the best of our knowledge, this is the first time that the expression level of long non-coding *sONE* was evaluated in a case–control study through collection of PE and normal placenta tissues.

Overall, our results suggest that perturbations in NO production and NO- related genes such as mir-155-5p may have a tight relation to the pathogenesis of PE. However, further studies are needed to confirm this fact. Since this study was performed on nulliparous women, the results obtained from our investigation could not be applied to all pregnant women. In some cases, probably using other hypothesesfor the pathogenesis of the disease could provide a comprehensive description for the risk factors associated with PE, but proving this requires further researches.

Based on the remarkable role in disease pathogenesis, designing drugs targeting NO production pathway, NO-related genes and biological reinforcing of NO could be a significant therapeutic strategy for patients. Moreover, further studies are warranted on NO production pathway and NO-related genes to more precisely characterize the molecular mechanisms involved in this disease.
